# Transgenerationally inherited incisor aberration mediated by stress in two isolated populations of *Microtus hartingi* vole

**DOI:** 10.1093/cz/zoag005

**Published:** 2026-02-04

**Authors:** Larisa Kaija, Tanya A Zorenko

**Affiliations:** Veterinary Public Health Institute, Animal Welfare Division, University of Bern, Länggassstrasse 120, Bern 3012, Switzerland; Faculty of Medicine and Life Sciences, University of Latvia, 1 Jelgava Street, Riga LV-1004, Latvia; Faculty of Medicine and Life Sciences, University of Latvia, 1 Jelgava Street, Riga LV-1004, Latvia

**Keywords:** arvicolinae, habitat fragmentation, social stress, transgenerational epigenetic inheritance, upper incisor overgrowth, vole

## Abstract

Environmental and social stress (SS) can alter phenotypic expression at both individual and population levels. We examined the effects of ancestral SS, imposed through forced polygyny, on reproductive success and maxillary incisor overgrowth in 2 isolated populations of *Microtus hartingi* vole: from Central Anatolia (CAP) and Eastern Rhodope (RP). In addition to ancestral SS, CAP voles were exposed to environmental stress during prenatal, lactation, and early-life periods. Under control conditions, the prevalence of incisor overgrowth was at a stable rate of ∼16.7% in RP and occurred very rarely (0.5%) in CAP. Stress exposure markedly increased prevalence in CAP but not in RP, independent of female kinship, ancestor sex, or generation. We propose that upper incisor overgrowth arises from an interaction between genetic background and stress-sensitive regulatory mechanisms, with stress releasing cryptic genetic variation. The RP, shaped by long-term habitat fragmentation, lack of dispersal, polygyny, and possibly inbreeding, appears to exhibit compensatory stabilization of this aberration, potentially through increased stress tolerance and earlier reproductive onset. CAP, originating from more stable environments, can be considered as a model of the early stage of RP adaptation to habitat fragmentation, as their heightened stress responsiveness leads to pronounced fluctuations in incisor overgrowth levels. Together, these findings suggest that stress-induced regulatory effects can persist across generations and may contribute to population divergence.

## Graphical Abstract

**Graphical Abstract:**
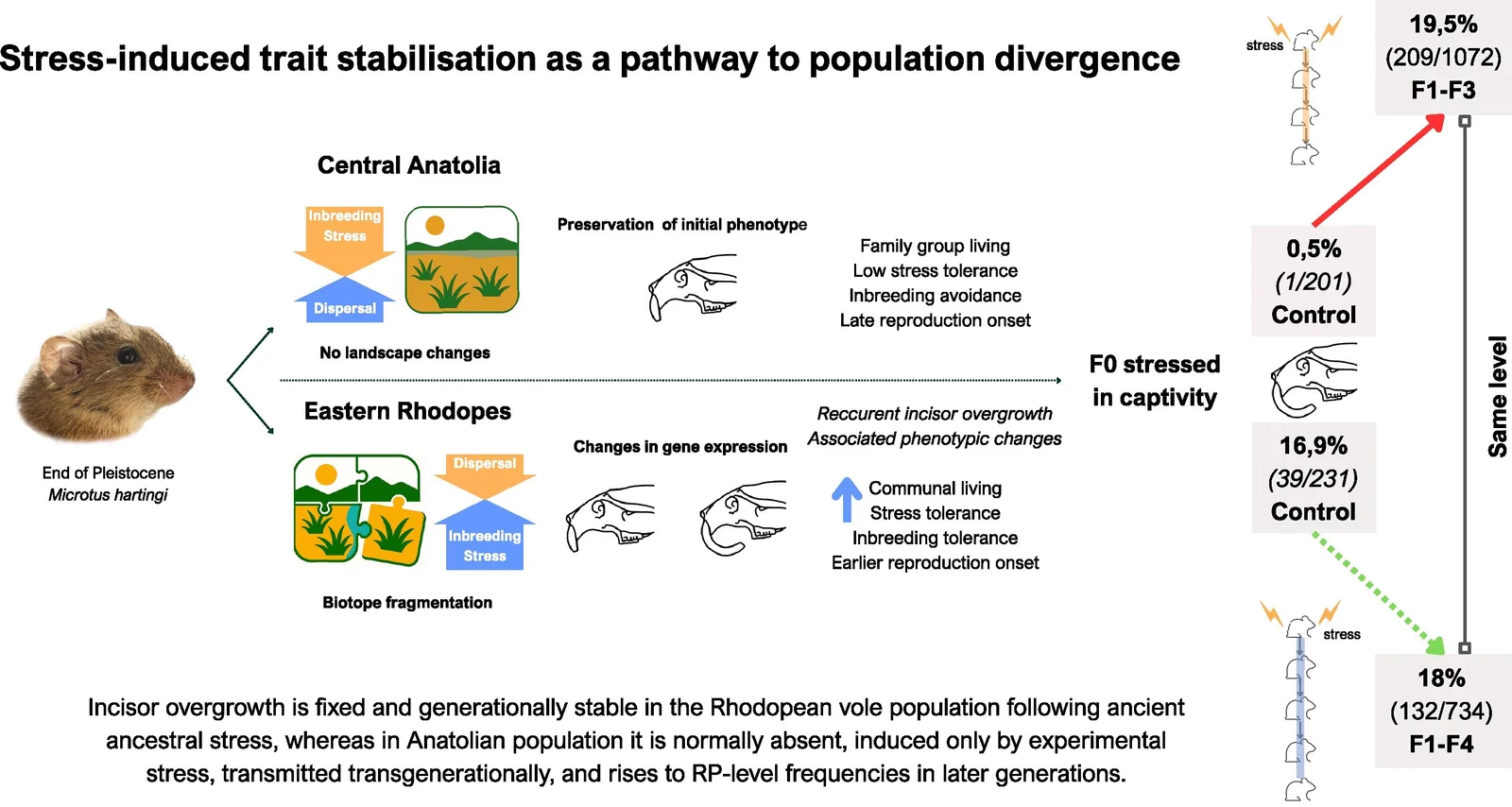

Craniofacial diversity is outstandingly high in rodents, reflecting their adaptability to various biomes and diets. The key feature of these mammals is the ability to phenotypically diverge over relatively short evolutionary timescales, which creates a high number of cryptic species and pioneer species able to survive under extreme environmental stress. Rodent rapid generational turnover makes them an effective model for examining adaptive radiation and the regulatory mechanisms shaping phenotypic variation.

Voles are characterized by hypselodont teeth, which undergo continuous eruption across the lifespan while preserving functional length. The emergence of hypselodont prismatic molars is thought to coincide with the aridification and spread of steppe-like biomes in the late Pliocene to early Pleistocene (Tesakov 2004), facilitating the efficient processing of abrasive vegetation in ancestral arvicolines.

Repeated climate and landscape changes throughout the Pleistocene and Holocene created highly unpredictable environments, a key factor in the evolution of modern species (Popov 2010). In such contexts, phenotypic plasticity is mediated by regulatory mechanisms that buffer or release underlying cryptic variation, enabling populations to respond rapidly to environmental fluctuations. Severe environmental stress can trigger the expression of previously hidden phenotypic variation, for example, through stress-responsive chaperone systems such as Hsp90 (Meiklejohn and Hartl 2002; Arnoldini et al. 2012; Guex 2016). Under unstable conditions, phenotypes, including dental morphology, may revert toward ancestral forms within only a few generations (Markova et al. 2020, 2023).

Environmental stress can be examined not only at the level of individual ontogeny or lineage inheritance, but also in the broader context of population-level adaptation and natural selection (Badyaev 2005; Hilbert 2013). Although ontogeny and evolution are intrinsically linked, the gene-centered framework of the Modern Synthesis traditionally assumes that processes acting during individual development do not influence phylogeny. This view has been increasingly challenged from an evo-devo perspective (Moore 2023; Pontarotti 2024). A growing body of evidence indicates that regulatory changes in gene expression can persist across multiple generations and may influence evolutionary trajectories, either directly or indirectly, forming the basis of the proposed Extended Evolutionary Synthesis (Tabadkani et al. 2012; Moore 2023; Sabaris et al. 2023; Osmanovic et al. 2025). Some authors further suggest that epialleles may function analogously to genetic alleles and thus become subject to natural selection, highlighting a potential interchangeability between genetic and environmental sources of heritable variation (Sabaris et al. 2023; Milocco and Uller 2024). Stress-induced regulatory changes can increase phenotypic variation without altering genotype, and repeated exposure to similar stressors may facilitate phenotypic accommodation and, occasionally, genetic assimilation (Harris et al. 2025; Badyaev 2005; Schlichting and Wund 2014; Lachmann and Jablonka 1996).

The relationship between stress-induced regulatory changes and mandibular or dental morphology remains largely unexplored. Mastication is physiologically linked to the hypothalamic–pituitary–adrenal axis, which regulates stress responses in mammals, and malocclusion can elicit both acute and chronic stress responses, potentially in a bidirectional manner (Ono et al. 2011). This suggests a link between stress responses and the emergence of aberrant dental phenotypes.

Dental and mandibular aberrations are commonly observed in both wild rodent populations (Jentzsch et al. 2020) and laboratory colonies (Basov and Zorenko 2025). Upper incisor elongation has been reported in several vole species, including *Mynomes californicus californicus*, *Mynomes californicus scirpensis*, *Myodes gapperi* (Imai et al. 2018), and *Microtus hartingi* (Zorenko, Basov, Kaijaet al. 2025). Molar apical elongation is likewise frequent in laboratory settings (Sugita et al. 1995; Jheon et al. 2015; Imai et al. 2018). Rodent dental aberrations are traditionally attributed to inadequate diet, especially the lack of abrasive material such as branches or gnawing sticks, resulting in abnormal chewing patterns (Soenjaya et al. 2015; Menegaz and Rasova 2017). Additional contributing factors include senescence (Dontas et al. 2010), trauma (About et al. 2000; Mancinelli and Capello 2016), and infection (Wiggs and Lobprise 1997). Heritable dental pathologies are comparatively understudied (Ida-Yonemochi et al. 2002; Wang et al. 2007; Imbschweiler et al. 2011; Soenjaya et al. 2015; Mancinelli and Capello 2016) and are often embedded within broader, complex phenotypic syndromes.

Mandibular and dental developmental abnormalities are rarely discussed in a regulatory context. Vole incisors are elodont and hypselodont, characterized by elongated crowns and open roots; in some species, true root formation does not occur at all (Legendre 2003; Wang et al. 2007). Dental cell differentiation, enamel and dentin secretion, mineralization, and stem cell proliferation are complexly regulated processes, partially occurring in the growth zone. Disruption of gene regulatory mechanisms can therefore result in aberrant dental phenotypes (Wang et al. 2007; Vogel et al. 2015). Amelogenesis alone involves the coordinated activity of more than 10,000 genes, and more than 115 associated pathologies have been described, arising from both genetic variation and gene–environment interactions (Yoshioka et al. 2015; Wright 2023).

Golenishchev et al. (2022) observed that skull thinning and molar apical elongation in voles can be associated with altered calcium–phosphorus metabolism. Heritable developmental aberrations of molars and incisors have been linked to gene–environment interactions in 2 subspecies of *Myodes californicus* (Gill and Bolles 1982). Prenatal processes contribute to a wide range of dental and craniofacial conditions, including molar and incisor hypomineralization (Garot et al. 2022), orofacial clefts (Dixon et al. 2011; Juriloff et al. 2014), pulpitis (Hui et al. 2017), temporomandibular joint osteoarthritis (Xiao et al. 2016), and chronic pain (Aya et al. 2012). DNA methylation has been implicated in pathologies of the temporomandibular joint, amelogenesis defects, bruxism, and a range of stress-related disorders involving the hypothalamic–pituitary–adrenal axis (Calic and Peterlin 2015).

DNA methylation and histone modification are the main drivers of intergenerational epigenetic inheritance (IEI). During germline development, primordial germ cells typically undergo genome-wide demethylation, followed by several waves of demethylation and remethylation before and after fertilization. The timing and extent of these reprograming events differ between maternal and paternal genomes. Nevertheless, some differentially methylated regions can escape reprograming and are transmitted to the embryo, a process known as genomic imprinting (Barlow and Bartolomei 2014). Consequently, methylation patterns present in parental gametes may be transmitted to offspring. When such effects persist beyond the third generation, they are classified as transgenerational epigenetic inheritance (TEI). By definition, TEI excludes direct gametic effects arising from exposure of the F0 female, which is often mediated through the paternal lineage, and can be influenced by factors such as diet, medicaments, and stress (Švorcová 2023).

In mammals, TEI appears to be less prevalent compared with IEI (Sabaris et al. 2023). During postnatal ontogenesis, the activity of DNA methyltransferases—enzymes that catalyze DNA methylation—decreases; however, their expression is still detectable in adult neurons (Feng et al. 2010). Chronic prenatal stress (PS) has been shown to induce changes in DNA methylation and histone acetylation in the prefrontal cortex and hippocampus of rats (Wei et al. 2021; Dion et al. 2022), is associated with depressive disorders in humans (Sales et al. 2020), and—together with early-life maternal separation and chronic social stress (SS) in adulthood—may have transgenerational consequences (Blaze and Roth 2015).

The role of chronic environmental stress in population-level adaptation, particularly with respect to dental morphotypes, remains an open question. To address this question, we studied 2 isolated populations of *M. hartingi* that differ in reproductive and social behavior (Zorenko 2022), levels of inbreeding tolerance (Zorenko and Kaija 2024), and the prevalence of incisor overgrowth and jaw deformities (Zorenko, Basov, Kagainis et al. 2025). Incisor overgrowth has been frequently observed in the Rhodopean population (RP) (Zorenko 2022); however, this aberration is absent in the *Microtus hartingi ankaraensis* population from Central Anatolia (CAP), both in wild-caught individuals and in laboratory-bred groups. In our laboratory population, CAP vole offspring showed a marked increase in the frequency of this phenotype after parental stress exposure.

Harting's vole occupies a range of mosaic habitats across southeastern Europe and Asia Minor. In Europe, its distribution is fragmented (Kryštufek et al. 2018; Zorenko, Basov, Kaija et al. 2025). The species entered southeastern Europe from Anatolia via a land corridor during the Middle Pleistocene (McHugh et al. 2008), and the divergence between the European (*M. hartingi* in Thrace) and Asian (*M. hartingi* in Anatolia) lineages occurred approximately 0.73 million years ago. Available evidence suggests that the reduction of steppe habitats, driven by the expansion of the Mediterranean mountain–forest complex in the Balkans (Okay and Okay 2002), resulted in periods of increased genetic drift and population bottlenecks in ancestral *M. hartingi* (Golenishchev et al. 2022). Consequently, habitat fragmentation, limited dispersal, and inbreeding imposed chronic stress on the Eastern RP, contributing to its divergence. Despite these harsh conditions, the RP did not go extinct but instead developed behavioral adaptations, including a communal reproductive strategy although maintaining high reproductive output (Zorenko 2022). In contrast, Central Anatolian voles typically live in family groups, have social monogamy, and actively avoid inbreeding. The RP shows reduced aggression and increased tolerance to inbreeding, independent of kinship structure (Zorenko and Kaija 2024).

Dental phenotypic aberrations have been reported in populations of *Dicrostonyx*, *Lemmus*, *Myopus*, and *Microtus* that experienced population bottlenecks under strong habitat fragmentation and genetic drift (Markova et al. 2020). Reduced heterozygosity has been proposed to decrease developmental robustness by limiting the complexity of gene regulatory networks, thereby increasing phenotypic instability (Hill et al. 2021).

Based on these findings, our primary hypothesis is that the high level of inbreeding in the RP (*M. hartingi*) was the underlying cause of incisor overgrowth. Our current hypothesis is that incisor overgrowth is triggered by the synergy of genetic factors and stress-induced epigenetic effects. The aim of this exploratory study is to investigate the role of stress in the transgenerational inheritance of incisor overgrowth. Specifically, we aim to: 1) evaluate the effect of different types of stress on the incisor overgrowth rates in offspring and 2) compare the incisor overgrowth patterns in 2 populations and to study the possible reasons behind the differences in the pattern.

## Materials and methods

### Model species

Laboratory populations of *M. hartingi* were established from founders sampled in 2 populations: the RP (*n* = 10 individuals) from the Eastern Rhodopes (Mandrica, Bulgaria; 41°41′N, 26°12′E) and the Central Anatolian population (*Microtus hartingi ankaraensis*, CAP; *n* = 8 individuals) from Kırşehir, Central Anatolia, Turkey (39°08′N, 34°09′E). Within each region, animals were caught from multiple localities. An additional cohort of CAP animals (7 males and 7 females, generation F5) was obtained from the Zoological Institute of the Russian Academy of Sciences (St. Petersburg, Russian Federation). Experimental work was conducted on RP individuals from generations F2–F4 and CAP individuals from generations F8–F11 at the Ethology Laboratory, University of Latvia. To maintain genetic variability during laboratory breeding, lineages were managed using a random mating scheme among unrelated individuals.

Animals were housed in cages (400 × 350 × 200 mm) with sawdust and hay provided as bedding and nesting material. To reduce the risk of incisor overgrowth and molar deformation, voles were supplied with paper tunnels as chewing substrates. During the winter–spring period, animals received woody branches as highly abrasive food. The standard diet included vegetables, apples, and a mixture of grain and hay. In spring and summer, fresh grass was provided. Experiments were conducted between March and December. Temperature was maintained at 20 ± 2 °C throughout the year, and animals were kept under a 12 h light:12 h dark photoperiod.

### Environmental stress (ES)

In 2016–2017, only CAP voles were assigned to 3 experimental groups subjected to different stress treatments and 1 control group (Figure 1). Reproductive success was assessed by recording litter size, pup survival to day 30, and the latency to the first litter. Offspring were weaned and transferred to separate cages at 35 days of age. At 60 days of age, individuals were paired to form new breeding pairs, producing 3 successive generations.

**Figure 1:**
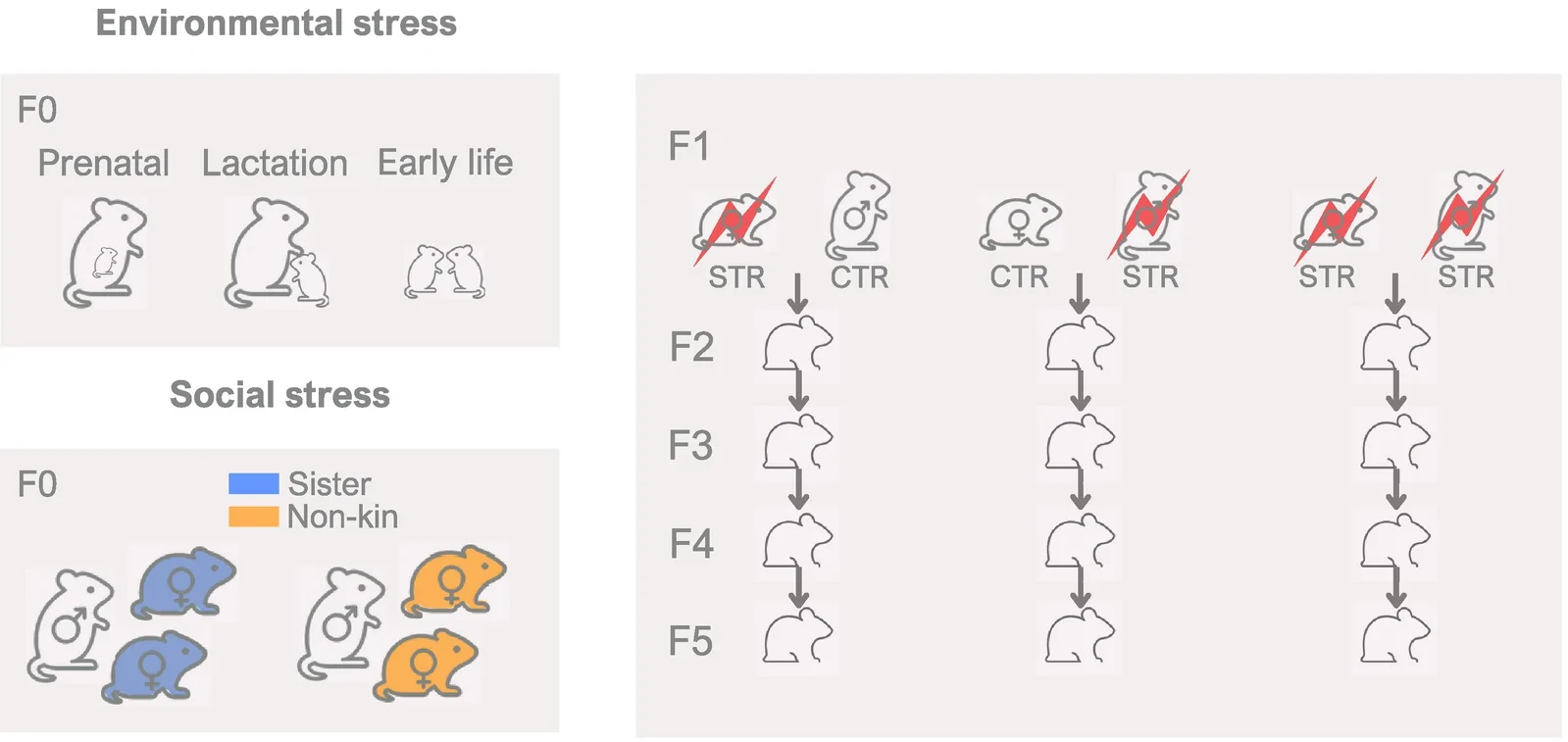
CAP animals were exposed to environmental stress: pregnant/lactating mother or newborn pups were placed in an empty container for 15 min/15 days. CAP and RP animals were exposed to SS: 2 sisters or non-kin females cohabited with a male in trios (F0). The offspring (F1) from different trios were paired either together (STR = stress) or with control (CRT) animals. The line was observed until F4.

1) *Prenatal stress*: From gestational days 5–7 (determined by maternal body mass gain), pregnant females were exposed to stress for 13–15 subsequent days (the duration of pregnancy is about 20 days). 2) *Lactation-related stress* (LS): lactating females were exposed to stress starting from the day of parturition. 3) *Early life stress* (ELS): pups were exposed to stress from the first postnatal day until eye opening at 10–13 days of age. 4) *Control group* (C): pairs lived under normal conditions. In the experimental stress groups, the female (PS and LS) or her pups (ELS) were placed for 15 min in a separate container with no bedding and the lid open. The temperature in the container was 5 °C lower than in the nest. After stress exposure, the voles were placed back to their home cages. Eight male-female pairs were assigned to each group, a total of 32 pairs.

### Social stress

Forced polygyny of both RP and CAP voles was applied in 2017–2019 (Zorenko 2022). Two SS treatments were applied: in the first (SSa), groups consisted of 2 sisters housed with an unrelated male; in the second (SSb), groups included 2 genetically unrelated females and a male. These groupings generated the following F1 offspring: 205 RP-SSa, 82 RP-SSb, 164 CAP-SSa, and 87 CAP-SSb. At 2 months of age, F1 individuals were paired for breeding.

Reproductive success was assessed as 1) the proportion of breeding females relative to the total number of females and 2) pup survival (number of surviving pups relative to the number born) until 30 days of age. The occurrence of upper incisor overgrowth was recorded as the proportion of affected individuals among all descendants. To assess parental transmission effects, F1 offspring from stressed F0 parents were paired for subsequent breeding under 3 conditions: SSa/b-B, in which both parents had experienced SS; SSa/b-M and SSa/b-F, in which only the male or the female parent had been stressed. Breeding success and the frequency of incisor overgrowth were monitored across generations up to F4–F5.

### Incisor overgrowth data systematization

Data on upper incisor overgrowth frequency were collected at the Ethology Laboratory of the University of Latvia between 2014 and 2022, as the laboratory populations were established and maintained. During data systematization and visualization, temporal patterns suggested an association between increased incisor overgrowth frequency and periods of experimental stress exposure (2016–2020) as well as inbreeding experiments (2020–2022) in both CAP and RP (Figure 2). These experiments were aimed at assessing the reproductive strategy of voles and tolerance to inbreeding/stress (Zorenko 2022; Zorenko and Kaija 2024).

**Figure 2:**
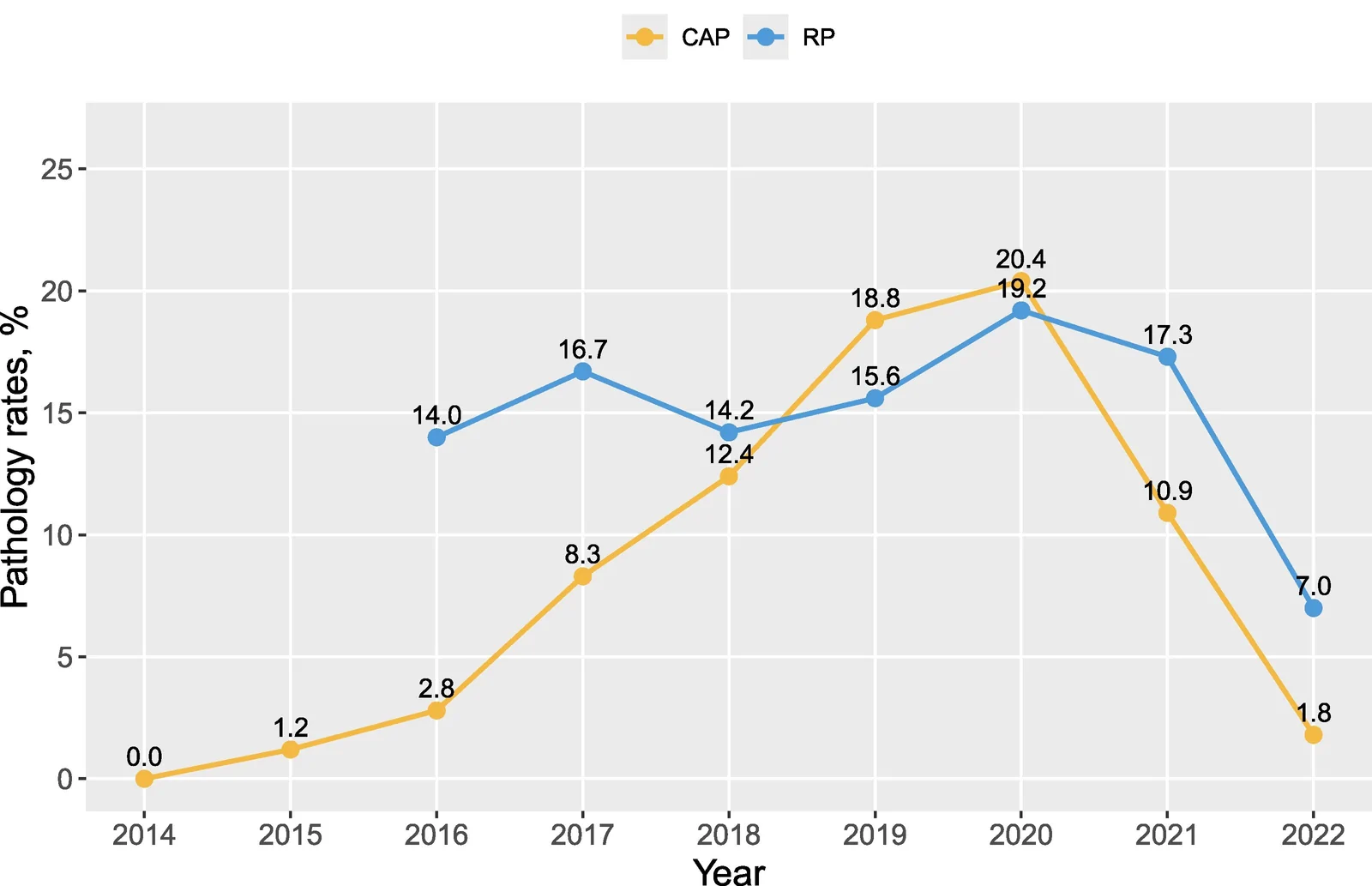
Temporal dynamics of maxillary incisor overgrowth in laboratory populations of RP (*n* = 1,438) and CAP (*n* = 1,643) voles after population establishment. Environmental stress tolerance experiments in CAP were conducted in 2016 and forced polygyny experiments involving co-housing of related and unrelated females were carried out between 2018 and 2020. A decline in the proportion of individuals exhibiting incisor overgrowth was observed in 2022, after the experiments on inbreeding (RP: 155/11 = 7.0%; CAP: 165/3 = 1.8%); however, the available data are insufficient to assess the specific contribution of inbreeding.

Our aim was to develop a model explaining how stress and inbreeding interact to shape divergent adaptive patterns in the 2 populations. Aberrant changes typically emerged early (on average after 4 months), allowing systematic data collection across all individuals. When the animals lost body mass and the ability to feed, they were sacrificed by cervical dislocation.

### Statistical analysis

Data were analyzed using R (version 4.1.1). Differences in frequencies of categorical outcome variables—including reproduction, offspring survival, and incisor overgrowth prevalence—were assessed using chi-square (χ²) tests. Comparisons were performed across generations, stress treatments, ancestor sex, and populations. Spearman's rank correlation was used to assess associations between incisor overgrowth prevalence, reproduction, and pup survival due to the bounded, non-normal nature of the data. We assessed whether incisor overgrowth rates were biased by the death of individuals before the typical age of overgrowth onset.

Incisor overgrowth was analyzed using a Bayesian binomial regression model, with the number of pathological incisors modeled relative to the total number of incisors per observation, fitted in R using the brms package. Population (CAP vs. RP), social condition (Control, SSa, and SSb), and their interaction were included as fixed effects to test for population-specific responses to SS. Generation was included as a centered continuous covariate to assess a monotonic transgenerational trend and was treated as numeric rather than categorical to avoid over-parameterization and to reflect gradual generational change. The analysis was conducted on the full dataset (40 observations) without aggregation; additional recorded variables were excluded because they would have introduced unnecessary model complexity given the available sample size. Weakly informative priors were used, convergence was assessed using R̂ and effective sample sizes, and results were reported on the probability scale in accordance with ARRIVE recommendations.

The graphical representations of methods (Figure 2) and the graphic abstract were made in Canva. Preparatory sketches were drawn in Procreate, then generated into icons (voles, steppe picture) in ChatGPT 4o, and finally refurbished in AdobePhotoshop. The photo of the vole (RP male) was taken in our lab. Generative AI was used to assist with scripts in R and partially as a tool for grammar check and translation to English.

## Results

### Environmental stress

#### Prenatal stress

Across 2 generations of control animals, reproductive success was stable, with a mean reproduction of 93.5% and a mean pup survival of 87.7%. Upper incisor overgrowth was rare, occurring in only 1 individual out of 201 animals (0.5%), born in generation F2.

In the PS stress group, 3 generations were obtained. Reproduction was lower in the second generation only (*P* < 0.001), whereas overall offspring survival was markedly reduced across all generations (*P* < 0.001), with no significant differences among generations (Table 1). The frequency of incisor overgrowth increased in all generations (*P* < 0.001) equally. Across generations, 7.9% of animals exhibited incisor overgrowth, representing an almost 16-fold increase relative to the control group.

**Table 1:** Effects of environmental stress on reproductive success and incisor overgrowth rates in CAP.

CAP	Generation	Reproduction		Offspring survival		Incisor overgrowth	
Control	1	19/20	95.0%	110/124	88.7%	0/96	0.0%
	2	23/25	92.0%	112/129	86.8%	1/105	0.95%
Prenatal stress	1	16/17	94.2%	61/90	67.8%	5/58	8.6%
	2	7/12	58.3%	36/54	66.7%	3/35	8.8%
	3	9/9	100.0%	33/59	55.9%	6/33	18.9%
Lactation stress	1	15/19	78.6%	47/87	54.0%	1/40	2.5%
	2	13/20	65.0%	53/74	71.6%	3/46	6.5%
	3	9/10	90.0%	33/45	73.3%	3/23	13%
Early life stress	1	9/10	90.0%	35/39	89.7%	5/33	15.2%
	2	8/11	72.3%	47/57	82.5%	1/34	2.9%
	3	5/5	100.0%	24/29	82.8%	2/19	10.5%

#### Lactation stress

Under LS, reproduction was reduced relative to controls (*P* = 0.003), and pup survival was even lower than under prenatal stress (PS; *P* < 0.001). Incisor overgrowth was low in F1 but increased in F2 (*P* = 0.003) and F3 (*P* < 0.001) compared with control. However, generation did not influence the results. Overall incisor overgrowth pattern under LS was similar to PS (Table 1); across all generations, 7.8% of animals were affected.

#### Early life stress

Reproduction and offspring survival were unaffected (Table 1). The pattern of incisor overgrowth differed from other types of environmental stress, as the highest prevalence occurred in the F1 generation (15.2%; *P* < 0.001), followed by a reduction to control levels in F2 and a subsequent increase in F3 (*P* = 0.0003). However, these fluctuations are likely influenced by the limited sample size. Upper incisor overgrowth prevalence under ELS differed significantly only from controls (*P* < 0.001). Across all generations, 9.4% of animals exhibited it.

Overall, environmental stress had minimal effects on reproduction but increased pup mortality—especially when mothers were stressed—and consistently elevated incisor overgrowth across treatments.

### Social stress

#### CAP. SSa (sister trios)

Three generations of polygynous CAP trios consisting of sisters were obtained. Reproduction was generally high (89.5%) among SSa groups defined by the sex of the stressed ancestor, although an escalation trend was observed in SSa-B compared with SSa-M (χ², *P* = 0.025). It did not differ across generations. SS markedly reduced offspring survival (Table 2). Survival was significantly lower than in controls in all SSa groups (uncorrected χ² tests, all *P* < 0.001), with the strongest reduction in SSa-M compared with SSa-B (χ², *P* = 0.033) and Ssa-F (χ², *P* = 0.003). A significant generational decline in survival was detected only in SSa-B (F2 vs. F1, *P* < 0.001). Upper incisor overgrowth was strongly elevated in all SSa groups compared with controls (uncorrected χ² tests, all *P* < 0.001) and did not differ significantly among SSa groups or between generations. Overall, SS in sister trios reduced offspring survival and increased incisor overgrowth.

**Table 2:** Effects of SS on reproductive success and incisor overgrowth rates in CAP and RP.

		Ancestor	Generation	Reproduction		Offspring survival		Incisor overgrowth	
CAP	Control		1	19/20	95%	110/124	88.7%	0/96	0.0%
			2	23/25	92%	112/129	86.8%	1/105	0.95%
	SSa	Both	1	12/12	100%	47/61	77.1%	9/41	21.95%
			2	9/9	100%	27/60	45.0%	7/27	25.9%
		Male	1	8/11	72.7%	33/66	50.0%	4/30	13.3%
			2	7/8	87.5%	22/52	42.3%	3/22	13.6%
		female	1	7/9	77.8%	28/37	75.7%	4/24	16.7%
			2	8/8	100%	48/71	67.6%	3/41	7.3%
	SSb	Both	1	20/31	64.6%	28/112	25.0%	3/23	13.0%
			2	6/7	85.7%	46/59	78.0%	13/41	31.7%
			3	7/8	87.5%	39/54	72.2%	4/42	9.5%
		Male	1	26/32	81.2%	78/163	47.9%	12/71	16.0%
			2	15/15	100%	71/122	58.20%	23/70	32.9%
			3	9/9	100%	31/45	68.9%	4/25	16.0%
		Female	1	17/23	73.9%	34/120	28.3%	5/25	20.0%
			2	2/4	50%	22/28	78.6%	6/21	28.6%
			3	7/7	100%	18/34	52.9%	4/18	22.2%
RP	Control		1	23/24	95.8%	163/194	84.0%	24/133	18.1%
			2	19/19	100%	140/153	91.5%	15/98	15.3%
	SSa	Both	1	20/21	95.2%	110/136	80.9%	11/77	14.3%
			2	14/15	93.3%	84/115	73.0%	10/59	16.95%
			3	14/14	100%	75/128	58.6%	11/52	21.2%
		Male	1	14/14	100%	113/122	92.6%	12/68	17.7%
			2	5/5	100%	19/23	82.6%	3/18	16.7%
			3	4/4	100%	17/30	56.7%	4/11	36.4%
		Female	1	13/13	100%	82/99	82.8%	10/56	17.9%
			2	6/6	100%	31/39	79.5%	2/20	10.0%
			3	4/4	100%	22/34	64.7%	2/20	10.0%
	SSb	Both	1	15/16	93.8%	78/124	62.9%	15/68	22.1%
			2	5/5	100%	32/40	80.0%	6/24	25.0%
			3	8/8	100%	34/50	68.0%	3/31	9.7%
			4	9/9	100%	37/43	86.1%	11/32	34.4%
		Male	1	8/8	100%	66/77	85.7%	6/33	18.2%
			2	8/9	88.9%	45/64	70.3%	5/44	11.4%
			3	10/10	100%	44/70	62.9%	10/36	27.8%
			4	3/3	100%	13/17	76.5%	4/13	30.8%
		Female	1	5/5	100%	17/27	62.96%	3/9	33.33%
			2	4/4	100%	30/31	96.77%	1/30	3.33%
			3	6/6	100%	45/61	73.77%	4/45	8.89%
			4	3/3	100%	21/32	65.62%	3/21	14.29%

#### CAP. SSb (unrelated trios)

In polygynous groups consisting of unrelated animals, 4 generations of offspring were obtained. Reproduction was high and stable across generations and did not differ from that observed in SSa. It showed no consistent dependence on the sex of the stressed F0 ancestor, with only isolated differences detected in individual generations. Offspring survival in SSb was significantly lower than in controls (χ², *P* < 0.001) and comparable with that observed in SSa. Survival was lowest in the F1 generation (51.8%) and increased significantly in subsequent generations, with no differences among F2, F3, and F4 (global χ² across generations, *P* ≈ 0.001). Survival characteristics varied transiently between groups defined by the sex of the stressed ancestor, without a stable sex-specific effect across generations.

Upper incisor overgrowth was markedly elevated relative to controls in SSb (χ², *P* < 0.001). Rates differed significantly between generations (global χ², *P* ≈ 0.007), peaking in the F3 generation (31.8%, 42/132), which represented the highest prevalence of incisor overgrowth observed in the experiment. No consistent directional trend across generations was evident.

Overall, SS had a strong effect on offspring survival and upper incisor overgrowth, with no consistent differences between SS types. Reproduction followed the pattern Control ≈ SSa ≈ SSb ≈ ELS > PS > LS (χ² tests, *P* < 0.02). Offspring survival followed Control ≈ ELS > PS ≈ LS ≈ SSa ≈ SSb (χ² tests, all *P* < 0.001). Incisor overgrowth followed the pattern SSb > SSa > ELS ≈ PS ≈ LS > Control, with all stress conditions differing from controls (χ², *P* < 0.001) and SSb showing higher rates than other stress conditions (χ² tests, *P* ≤ 0.02). The sex of the stressed ancestor had no consistent effect on reproduction, offspring survival, and upper incisor overgrowth.

Thus, SS had a particularly strong effect on incisor overgrowth, whereas environmental stress primarily reduced reproductive function of animals.

#### RP. SSa (sister trios)

In the RP, control groups showed consistently high reproduction (mean 97.7%) and offspring survival (mean 87.3%), comparable with CAP controls (Table 2). Upper incisor overgrowth, however, was substantially more prevalent in RP controls (16.9%, 39/231) than in CAP controls (0.5%; χ², *P* < 0.001).

Across 4 generations of RP sister trios, reproduction remained uniformly high (95.2–100%) and was not dependent on the generation or sex of the stressed ancestor. On the contrary, the survival of offspring in subsequent generations decreased compared with the control group: survival in F1 did not differ from controls (χ², *P* = 0.466), in F2, a significant decrease was observed (χ², *P* < 0.001), whereas in F3, a pronounced drop occurred (χ², *P* < 0.001).

Survival differed between sex-defined stress groups in F1 (χ² = 7.87, *P* = 0.0195), but not in the F2 (*P* = 0.511) or F3 (χ², *P* = 0.295). Whereas survival in F1 was highest after male-line stress, the lowest survival values in later generations were observed in the both-parent–stressed group. Accordingly, a strong generational decline was evident in the both-parent–stressed group (χ² = 16.24, *P* < 0.001), indicating transient and non-directional sex-specific effects rather than a stable sex-of-origin influence. Incisor overgrowth frequencies in RP sister trios did not differ significantly from RP controls, although descriptive variation among stress groups was observed (Table 2).

Relative to CAP sister trios, RP sister trios exhibited higher reproduction (97.8% vs. 89.5%; χ², *P* = 0.027) and higher offspring survival (77.2% vs. 59.1%; χ², *P* < 0.001), whereas incisor overgrowth under stress did not differ between populations (χ², *P* = 0.801).

#### RP. SSb (unrelated trios)

Five generations were obtained from F0 trios consisting of unrelated females. Reproduction remained high under both sister (SSa) and unrelated (SSb) SS and did not differ from control levels, showing no consistent dependence on the generation or sex of the stressed ancestor. Offspring survival was reduced relative to controls (χ², *P* < 0.001) but did not differ between SSa and SSb.

In SSb, offspring survival varied depending on the sex of the stressed ancestor only in early generations. In F1, survival was highest in male-stressed groups (χ², *P* ≤ 0.02), whereas in F2 it was highest in female-stressed groups (χ², *P* ≤ 0.04). Beginning with the F3, no significant sex-specific differences were detected, indicating that the sex effect on offspring survival was transient and not maintained across generations (Table 2). Upper incisor overgrowth frequencies in RP SSb were comparable with those in RP controls and showed irregular, non-systematic variation across generations and sex-defined groups.

Overall, SS affected offspring survival but had little effect on reproduction or incisor overgrowth in the RP. In both populations, reproduction followed the relationship: Control ≈ SSa ≈ SSb, indicating resilience to SS. Offspring survival followed: Control > SSa ≈ SSb, but survival was consistently higher in RP than in the CAP. Incisor overgrowth showed a stress-dependent pattern (SSb > SSa > Control) only in CAP, whereas RP showed: Control ≈ SSa ≈ SSb, indicating population-specific sensitivity to SS.

Across all observations, upper incisor overgrowth prevalence showed a weak negative association with pup survival (Spearman's *ρ* = −0.18, *n* = 40, *P* = 0.256), while reproductive success showed a weak positive association with pup survival (*ρ* = 0.22, *n* = 40, *P* = 0.168). When analyzed separately, the associations remained weak and statistically insignificant in both populations (CAP: incisors/survival *ρ* = −0.12, *P* = 0.660; reproduction/survival *ρ* = 0.15, *P* = 0.568; RP: incisors/survival *ρ* = −0.24, *P* = 0.275; reproduction–survival *ρ* = 0.09, *P* = 0.668).

### Hierarchical model

Rates of incisor overgrowth depended strongly on both population background and stress exposure (Supplementary Table S1). Under control conditions, the RP exhibited substantially higher incisor overgrowth prevalence than the CAP (posterior probability RP > CAP = 1.00). In CAP animals, both SS conditions markedly increased the probability of incisor overgrowth relative to controls (SSa > Control = 1.00; SSb > Control = 1.00). In contrast, although stress also increased overgrowth in RP animals, these effects were weaker and more variable (SSa > Control: 0.68; SSb > Control: 0.78).

A significant interaction between population and social condition revealed that the stress-related increase in incisor overgrowth was significantly reduced in RP individuals compared with CAP. Thus, although RP animals exhibited higher baseline levels of overgrowth, CAP animals were significantly more sensitive to SS. Generation, included as a continuous predictor, showed no clear monotonic effect on incisor overgrowth. The estimated generation slope was small, and its confidence interval overlapped zero, indicating little evidence of a systematic increase or decrease in pathology across generations (Figure 3). Posterior contrasts comparing early and late generations yielded moderate probabilities (≈0.65) that pathology increased over time, but these effects were weak relative to the pronounced population and stress effects and were consistent across conditions due to the absence of generation-specific interactions.

**Figure 3:**
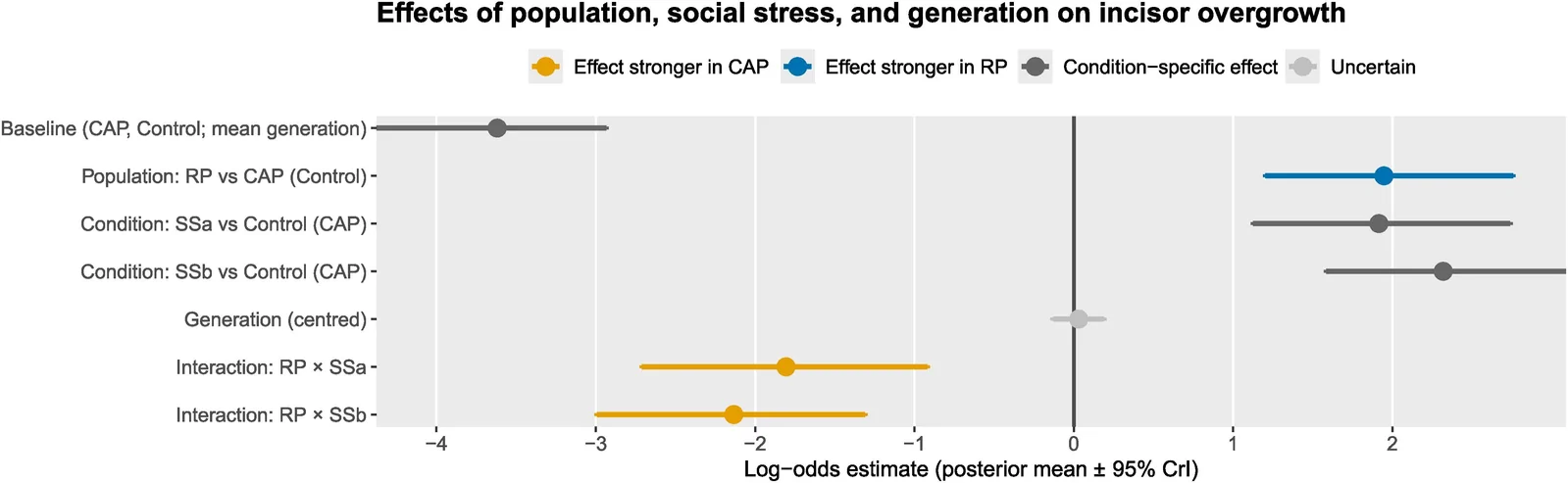
Effects of population, SS, and generation on incisor overgrowth. Posterior mean log-odds estimates from a Bayesian binomial model are shown with 95% credible intervals. Effects are expressed relative to the CAP control group at the mean generation. Positive values indicate higher incisor overgrowth probability; negative values indicate lower probability. Colors indicate whether effects are stronger in CAP or RP, condition-specific, or uncertain (credible interval overlapping zero). The vertical line denotes no effect.

Model-based predictions indicated a higher prevalence of incisor overgrowth in the RP (posterior mean 17.6%, 95% CrI 14.9–20.6%) than in the CAP (13.5%, 95% CrI 10.9–16.5%), averaged over generations and social conditions. Overall, incisor overgrowth was primarily determined by population background and SS exposure, with strong population-specific stress sensitivity but with little evidence of cumulative transgenerational change in this trait.

The general analysis suggests that rates of incisor overgrowth are higher and more stable in RP, while in CAP it is mostly triggered by stress (Figure 3).

## Discussion

Upper incisor overgrowth has been previously reported in 2 populations of Harting's vole (*Microtus hartingi strandzensis* and *M. hartingi* from the Rhodope Mountains), but not in *M. h. ankaraensis* (CAP), either in the wild or under long-term laboratory maintenance (e.g., in laboratory colonies of LU and ZIN). However, our results demonstrate that upper incisor overgrowth can be induced in CAP under certain environmental and SS conditions. Unlike RP, where incisor overgrowth was observed with a stable frequency regardless of experimental manipulations, CAP exhibited a pronounced stress-dependent response, with particularly strong effects under SS involving unrelated females.

After applying stress manipulations to pregnant and lactating females, as well as to offspring during the lactation period, 2.5–18.2% of descendants exhibited a modified phenotype characterized by growth of the maxillary incisors (Table 2). Importantly, this phenotype did not disappear in subsequent generations (F2–F5) but persisted at the same rate, with an average frequency of approximately 10%.

In the RP, incisor overgrowth was observed at a consistently high frequency, with similar prevalence in both control (no stress) and experimental (SS) groups with insignificant fluctuations. Data from established groups of related and unrelated females in the RP (Zorenko and Kaija 2024) indicate that social factors had little or no detectable influence on the occurrence of this aberration. Across generations, the proportion of affected individuals remained stable at approximately 17.8%, and no effect of the sex of the stressed ancestor was observed.

SS had an even stronger effect on incisor overgrowth in *M. h. ankaraensis*, as its prevalence approximately doubled (to ∼18%) compared with environmental stress alone. This increase was greater in groups of unrelated females in CAP (SSb), highlighting the importance of social novelty as a potent developmental stressor.

Incisor overgrowth showed a stress-dependent pattern (SSb > SSa > Control) only in CAP, whereas RP showed Control ≈ SSa ≈ SSb, indicating population-specific sensitivity to SS. These interpretations are supported by the hierarchical Bayesian model, which showed a strong population × SS interaction, with markedly attenuated stress effects in RP despite higher baseline prevalence. Notably, the level of incisor overgrowth under stress was comparable between the RP, which has a long evolutionary history, and *M. h. ankaraensis*, in which a transition to communal breeding was induced experimentally over a relatively short timescale (χ² = 0.18, df = 1, *P* = 0.67).

The maintenance of the phenotype over several generations in the absence of direct stress exposure suggests transgenerational inheritance, probably caused by methylation and histone acetylation (Hunter and McEwen 2013), which would be important to elucidate in the future. Apparently, the Rhodope vole has undergone stabilization of this phenotype, which is consistent with the process of genetic assimilation. However, this cannot be stated for CAP, because the phenotype may not manifest in subsequent generations or be rare and random. Isolated individuals with overgrown incisors have been reported across a wide range of murine rodents in the wild (Jentzsch et al. 2020; A. Basov, pers. comm.), where such cases likely represent direct environmental responses. In the Rhodopean vole, however, the trait appears to have become heritable and transmitted across generations.

Incisor overgrowth in both populations of Harting's voles typically manifests at around 4 months of age (Zorenko, Basov, Kagainis et al. 2025), with the earliest cases observed in 2-month-old individuals and the latest cases occurring between 8 and 10 months of age. In the present study, reproductive success and pup mortality were explicitly controlled to ensure that potentially affected individuals did not die before the age at which the aberration usually becomes apparent. Although overgrowth rates increased with mortality, the association was weak (CAP: *ρ* = −0.12; RP: *ρ* = −0.24). The late onset of incisor overgrowth allows individuals to reproduce before the development of dental abnormalities that substantially reduce the quality of life and ultimately lead to death.

Reproductive data for *M. h. ankaraensis*, obtained from both wild populations (Yiğit and Çolak 2002) and laboratory studies (Zorenko 2022) indicate that this subspecies is characterized by social monogamy, avoidance of inbreeding, and low tolerance to SS (Zorenko and Kaija 2024). In earlier experimental studies, polygynous trios consisting of related or unrelated females were created; however, reproduction typically occurred in only 1 female (Zorenko and Kaija 2024). When both females reproduced, most offspring died shortly after birth. Surviving pups developed under unfavorable conditions associated with heightened maternal excitability, loss of lactation, and reduced milk quality (Gromov 2011). Daughters of such mothers often demonstrated reduced parental care, leading to subsequent offspring mortality. These processes may contribute to the intergenerational transmission of stress-induced effects. Nevertheless, in the present study, we did not find higher incisor overgrowth rates in groups obtained from stressed females compared with groups obtained from non-stressed males.

In contrast, in experimental groups of Rhodope voles, SS had little effect on reproduction and only moderately increased mortality relative to CAP groups (Table 2). RP voles experience less stress when housed with sisters and generally exhibit high tolerance to SS (Zorenko and Kaija 2024). The weak effect of experimentally induced SS in this population is consistent with the fact that the transition from social monogamy to communal breeding occurred thousands of years ago, representing an evolutionary stage that *M. h. ankaraensis* appears to be entering only under experimental conditions. The social strategy of Rhodope voles includes living in larger social groups, communal breeding, periodic cross-fostering of offspring, larger litters, and higher offspring survival (Zorenko 2022), traits that can be considered key adaptations facilitating successful colonization of new territories in Europe.

Within a threshold framework of trait expression, incisor overgrowth can be interpreted as a quantitatively regulated trait that becomes phenotypically expressed only when underlying genetic and epigenetic variation exceeds a critical threshold. In CAP, this threshold is apparently not reached under normal conditions, whereas in RP it has probably been historically exceeded.

This interpretation is consistent with signal theory, which posits that signal intensity must exceed background environmental noise to elicit a phenotypic response. Epigenetic signals can be incorporated into this framework, as they encode environmental information in the form of DNA methylation marks that subsequently regulate phenotypic outcomes. Genetic factors, including cryptic genetic variation, in turn influence the efficiency with which such epigenetic signals are transduced (Laubach et al. 2018).

Empirical evidence supports a genetic contribution to the stability of epigenetic marks. Temporal changes in DNA methylation, likely including stochastic changes due to drift, are more similar within families than between families, suggesting a heritable component in the accumulation of epigenetic errors (Bjornsson et al. 2008). Gill and Bolles (1982) proposed that such mechanisms underlie the inheritance of molar overgrowth in *My. californicus*. In European populations of *M. hartingi* from the Rhodope Mountains (RP) and *M. h. strandzensis*, the threshold for incisor overgrowth appears to have been reached (Zorenko, Basov, Kaija et al. 2025), whereas in *M. h. ankaraensis* an additional provoking factor—environmental or SS—is required for the phenotype to manifest.

Grüneberg (1963) suggested that small, discrete skeletal variations may serve as indirect markers of genetic differentiation among mammalian populations, as their frequencies correlate with major mutations in different mouse strains. He demonstrated that many traits have an underlying quantitative basis but are phenotypically expressed only when a threshold is exceeded. Falconer (1965) formalized this principle in a multifactorial threshold model for disease susceptibility. Berry and Searle (1963) showed that populations of wild house mice exhibit the same non-metric skeletal traits as laboratory strains, supporting the view that such traits reflect latent and threshold-dependent quantitative variation. Consistent with this framework, epigenetic divergence index estimates based on nonmetric cranial traits revealed marked differentiation in voles from the south-eastern Balkan Peninsula (*M. h. strandzensis*), but not between *Microtus hartingi lydius* and *M. h. ankaraensis* (Markov et al. 2014). Significant variations were also detected in Harting's voles, including the overgrowth of the upper incisors and molars, shortening of the lower incisors, and deformation of the lower jaw (Zorenko, Basov, Kagainis et al. 2025). Moreover, similar novel phenotypes were observed in both the RP and the CAP, in which all dental changes were induced by experimental stress.

Cryptic genetic variation is thought to accumulate through drift and remain phenotypically silent until released by perturbing influences. Under severe environmental or SS, previously dormant traits may become expressed (Masel and Trotter 2010; Iwasaki et al. 2013). In the subfamily Arvicolinae, increased dental diversity—particularly crown complexity and irregular occlusal patterns—has been reported under conditions of inbreeding and population bottlenecks (Markova et al. 2020). The RP is thought to have experienced a bottleneck (Golenishchev et al. 2022) and has persisted for at least 10,000 years in a highly fragmented habitat (Zorenko, Basov, Kaija et al. 2025) under chronic environmental stress. Under such conditions, selection likely favored individuals with increased tolerance to stress and inbreeding.

Over many generations, these processes may have led to the stabilization of incisor overgrowth in RP through epigenetic rearrangement initiated by chronic environmental stress (Lachmann and Jablonka 1996; Chapelle and Silvestre 2022; Markova et al. 2023). The persistence of incisor overgrowth, despite its deleterious nature, suggests that population survival depended on compensatory advantages, including communal reproduction, high reproductive output without dispersal, early reproduction prior to the onset of incisor overgrowth (Zorenko 2022), and increased tolerance to inbreeding.

Comparative data support this interpretation. In *M. h. strandzensis* captured in the late 1980s, incisor overgrowth was not observed during 12 years of laboratory breeding (Zorenko, Basov, Kaija et al. 2025), whereas in individuals collected between 2004 and 2012, incisor overgrowth and jaw deformation were repeatedly recorded, presumably reflecting increasing habitat fragmentation (Markov and Dimitrov 2010). Similarly, incisor and molar aberrations are widespread among Nearctic vole species (Harvey et al. 2009; Imai et al. 2018). Amargosa voles (My. *c. scirpensis*), which inhabit an extremely fragmented and isolated habitat, exhibit molar aberrations in up to 63% of individuals under laboratory conditions (Imai et al. 2018), alongside early sexual maturity and communal breeding strategies comparable with those of RP voles.

Model-based predictions indicated that the prevalence of incisor overgrowth was below than the 25% expected under a simple autosomal recessive model with complete penetrance (Aa × Aa) in both populations (CAP: 13.5%, 95% CrI 10.9–16.5%; RP: 17.6%, 95% CrI 14.9–20.6%), suggesting incomplete penetrance and/or a more complex genetic and environmental basis. However, unpublished breeding data indicate that in many *M. hartingi* pairs approximately 25–30% of offspring are affected, whereas in some families the overgrowth is expressed in either all or none of the progeny, consistent with segregation patterns expected for a single-gene Mendelian effect.

Experimental inbreeding aimed at assessing purging effects produced inconclusive results due to limited sample size; however, F2 individuals derived from stressed lines no longer expressed the incisor overgrowth (Supplementary Table S2a and 2b). In the isolated RP, alternating periods of inbreeding and outbreeding may have intensified natural selection, favoring high tolerance to inbreeding (Zorenko and Kaija 2024). Such tolerance may represent an adaptive trait contributing to the maintenance of incisor overgrowth at a low but stable frequency.

Taken together, these findings suggest that *M. h. ankaraensis* represents a promising model for investigating how stress-induced incisor overgrowth may become stabilized and potentially assimilated over evolutionary time in the RP.

## Authors’ contributions

Conceptualization, L.K. and T.A.Z.; methodology, T.A.Z. and L.K.; formal analysis, L.K., investigation, T.A.Z.; writing—original draft preparation, L.K. and T.A.Z.; writing—review and editing, T.A.Z. and L.K.; funding acquisition, L.K. and T.A.Z.; collecting material on voles in the field, T.A.Z. All authors have read and agreed to the published version of the manuscript.

## Supplementary Material

zoag005_Supplementary_Data
