# Supplementary material for: Transgenerationally inherited incisor aberration mediated by stress in two isolated populations of Microtus hartingi vole
Source: Curr Zool. 2026 Feb 4;72(5):747–58. doi: 10.1093/cz/zoag005 (PMC13690578; doi:10.1093/cz/zoag005)
Supplement: Supplementary file 1 [file zoag005_supplementary_data.docx]

**Supplementary Data**

*Supplemetary table 1. Inbreeding effects in CAP vole generations after stress.*

| *incisor_overgrowth \| trials(incisor_total) ~ population * condition + gen_c (40 obseravtions)* | | | | | | | |
| --- | --- | --- | --- | --- | --- | --- | --- |
|  | Regression Coefficients | | | | | | |
|  | **Estimate** | **Est.Error** | **l-95% CI** | **u-95% CI** | **Rhat** | **Bulk_ESS** | **Tail_ESS** |
| Intercept | -3.62 | 0.37 | -4.09 | -2.93 | 1.00 | 2143 | 3410 |
| populationrp | 1.95 | 0.40 | 1.20 | 2.76 | 1.00 | 2105 | 3427 |
| conditionSSa | 1.92 | 0.41 | 1.13 | 2.74 | 1.00 | 2414 | 3461 |
| conditionSSb | 2.32 | 0.39 | 1.58 | 3.09 | 1.00 | 2278 | 3567 |
| gen_c | 0.03 | 0.08 | -0.13 | 0.19 | 1.00 | 4447 | 4433 |
| populationrp:conditionSSa | -1.81 | 0.45 | -2.71 | -0.92 | 1.00 | 2380 | 3626 |
| populationrp:conditionSSb | -2.13 | 0.43 | -2.99 | -1.31 | 1.00 | 2287 | 3435 |

**Intercept.** Baseline log-odds of incisor overgrowth for the reference group: CAP population, control condition, at the mean-centered generation.

**populationRP.** Difference in log-odds of incisor overgrowth between the Rhodopean population (RP) and CAP, under control conditions.

**conditionSSa**. Effect of social stress with sisters (SSa) relative to control, in the CAP population.

**conditionSSb.** Effect of social stress with unrelated females (SSb) relative to control, in the CAP population.

**gen_c.** Effect of generation number, treated as a continuous, mean-centered predictor, representing change in incisor overgrowth across generations.

**populationRP:conditionSSa.** Interaction term indicating how the effect of SSa differs in RP compared with CAP.

**populationRP:conditionSSb.** Interaction term indicating how the effect of SSb differs in RP compared with CAP.

*Supplementary table 2a. Inbreeding effects in CAP vole generations after stress.*

| *M. h. ankaraensis* (CAP) | | | | | | | | | | | |
| --- | --- | --- | --- | --- | --- | --- | --- | --- | --- | --- | --- |
| Control | F1 | F2 | Inbreeding | F0 | F1 | F2 | F3 | One inbred parent | Total | Two  inbred parents | Total |
| *Reproduction* | 19/20  94.4% | 23/25 92% |  | 10/10 100% | 10/10 100% | 12/12 100% | 2/3  100% |  | 6/6 100% |  | 16/16 100% |
| *Offspring survival* | 110/124 88.7% | 112/129 86.8% |  | 47/53 88.7% | 74/83 89.2% | 62.78 79.5% | 8/9 88.9% |  | 46/50 92% |  | 72/94 76.6% |
| *Incisor*  *overgrowth* | 0/96  0% | 1/105 0.95% |  | 8/47 17% | 2/67 2.98% | 1/42 2.38% | 0/8  0% |  | 0/37 0% |  | 3/66 4.5% |

*Supplementary table 2b. Inbreeding effects in RP vole generations after stress.*

| *Microtus hartingi* (RP) | | | | | | | | | | | | |
| --- | --- | --- | --- | --- | --- | --- | --- | --- | --- | --- | --- | --- |
| *Control* | F1 | F2 | Inbreeding | F0 | F1 | F2 | F3 | One inbred parent | Male | Female | Two inbred parents | Total |
| *Reproduction* | 23/24 95.8% | 19/19 100% |  | 28/30 93.3% | 21/21 100% | 18/18 100% | 10/10 100% |  | 7/7  100% | 26/26 100% |  | 16/16 100% |
| *Offspring*  *survival* | 163/194 84% | 140/153 91.5% |  | 122/140 87.1% | 100/129 77.5% | 80/99 80.8% | 42/48 87.50% |  | 34/41 82.9% | 116/141 82.3% |  | 76/103  73.7% |
| *Incisor overgrowth* | 24/133 18% | 15/98 15.3% |  | 14/67 20.9% | 15/75 20% | 1/69 1.5% | 0/25  0% |  | 0/19 0% | 11/87 12.6% |  | 2/66  3% |
